# Mucocutaneous‐Predominant Pediatric Behçet's Disease With Recurrent Oral and Genital Ulceration: A Case Report

**DOI:** 10.1002/ccr3.73068

**Published:** 2026-06-30

**Authors:** Fares Basel Abu Taha, Alaweya A. Ofash, Osama S. Abbadi, Faris Abdon, Adil Khalil Hussien Khalil, Abdelrahman M. A. Abukanna, Amani M. Nabri, Mohammed Moizulddin Khan, Mahir Mohammed

**Affiliations:** ^1^ Department of Internal Medicine Red Sea University Port Sudan Sudan; ^2^ Department of Biochemistry, Faculty of Medicine The National University Khartoum Sudan; ^3^ Department of Medical Sciences Orotta College of Medicine and Health Sciences Asmara Eritrea; ^4^ Basic Medical Sciences Department, College of Medicine Dar Al Uloom University Riyadh Saudi Arabia; ^5^ Clinical Sciences Department, College of Medicine Dar Al Uloom University Riyadh Saudi Arabia; ^6^ Dermatology Department Fujairah Hospital Fujairah UAE

**Keywords:** Behçet's disease, colchicine, genital ulcer, oral ulcer, pathergy test, pediatric

## Abstract

A 12‐year‐old boy presented with recurrent oral and scrotal ulcers, papulopustular lesions, arthralgia, and a positive pathergy test. Autoimmune tests were negative; ophthalmologic and gastrointestinal assessments were reassuring. Topical therapy and colchicine produced sustained improvement. This case supports early recognition and surveillance in mucocutaneous‐predominant pediatric Behçet's disease.

## Key Clinical Message

Key Clinical MessageA supportive pathergy test in a child with recurrent oral and genital ulceration, papulopustular lesions, and arthralgia can strengthen early recognition of mucocutaneous‐predominant Behçet's disease and guide structured surveillance for later major‐organ involvement.

**Abbreviations**
ALP: alkaline phosphatase
ALT: alanine aminotransferase
ANA: antinuclear antibody
AST: aspartate aminotransferase
BD: Behçet's disease
ENA: extractable nuclear antigens
ESR: erythrocyte sedimentation rate
GI: gastrointestinal
HLA: human leukocyte antigen
MCV: mean corpuscular volume
PEDBD: Pediatric Behçet's Disease (criteria)
tTG‐IgA: tissue transglutaminase IgA
WBC: white blood cell count

## 1 Introduction

Behçet's disease is a chronic, relapsing, multisystem variable‐vessel vasculitis characterized mainly by recurrent oral aphthous ulceration, with genital ulcers, papulopustular or erythema nodosum‐like skin lesions, ocular inflammation, and musculoskeletal involvement occurring in many patients, and gastrointestinal, neurologic, or vascular disease in a subset [1, 2, 3]. Pediatric‐onset disease is well recognized, but diagnosis is often delayed because manifestations may appear gradually rather than together early in the course of illness [4, 5]. In the prospective PEDBD cohort, oral aphthosis was the presenting feature in most children, and the mean delay to a second manifestation was 2.9 ± 2.2 years, illustrating the stepwise evolution that can hinder early recognition [6].

Diagnosis remains clinical because there is no single pathognomonic laboratory or histopathologic test [7]. The International Criteria for Behçet's Disease are useful in practice because they incorporate oral and genital aphthosis, ocular and skin manifestations, neurologic and vascular disease, and an optional pathergy test in a point‐based system [8]. In children, however, early disease may be incomplete, which is why pediatric‐specific frameworks such as the PEDBD criteria are helpful [6]. The pathergy test is also a useful supportive bedside finding when interpreted in the context of a compatible phenotype, although positivity rates vary across populations [2, 9].

Mucocutaneous manifestations are particularly important in pediatric Behçet's disease because they are often the dominant features at presentation, while major‐organ involvement may emerge later [10, 11]. This makes structured surveillance essential even when the initial phenotype appears limited. Ocular involvement is clinically important because it may threaten vision over time [12], while gastrointestinal, neurologic, and vascular disease account for substantial morbidity in more severe cases [13, 14].

This case illustrates how recurrent oral and genital ulceration with compatible skin lesions, arthralgia, and a positive pathergy test can support early diagnosis of pediatric Behçet's disease before major‐organ involvement becomes evident. It also underscores the need for continued ophthalmic, gastrointestinal, neurologic, and vascular surveillance after the initial diagnosis [2, 6, 15].

## 2 Case History/Examination

A 12‐year‐old Jordanian boy with no known chronic illness presented with recurrent painful oral ulceration for more than 12 months, occurring approximately monthly, together with episodic painful scrotal ulceration. During the same period, he reported intermittent arthralgia affecting the hands, feet, and knees, more pronounced on the right side, without swelling, redness, or restriction of movement. He also described fatigue, episodic tender cervical lymphadenopathy, and intermittent abdominal discomfort with nausea and occasional diarrhea. He denied visual loss but reported intermittent ocular itching. His past medical history was notable for a pulmonary hydatid cyst successfully treated about 2 years earlier. This previous hydatid disease was considered clinically incidental and was not thought to explain the later Behçet‐compatible inflammatory presentation.

There was no known family history of Behçet's disease, recurrent oral or genital ulceration, or inflammatory bowel disease. Given the genital ulceration, infection‐related and safeguarding concerns were reviewed clinically. There was no history suggestive of sexual exposure or abuse, and the overall pattern favored a non‐infectious inflammatory disorder.

**FIGURE 1:**
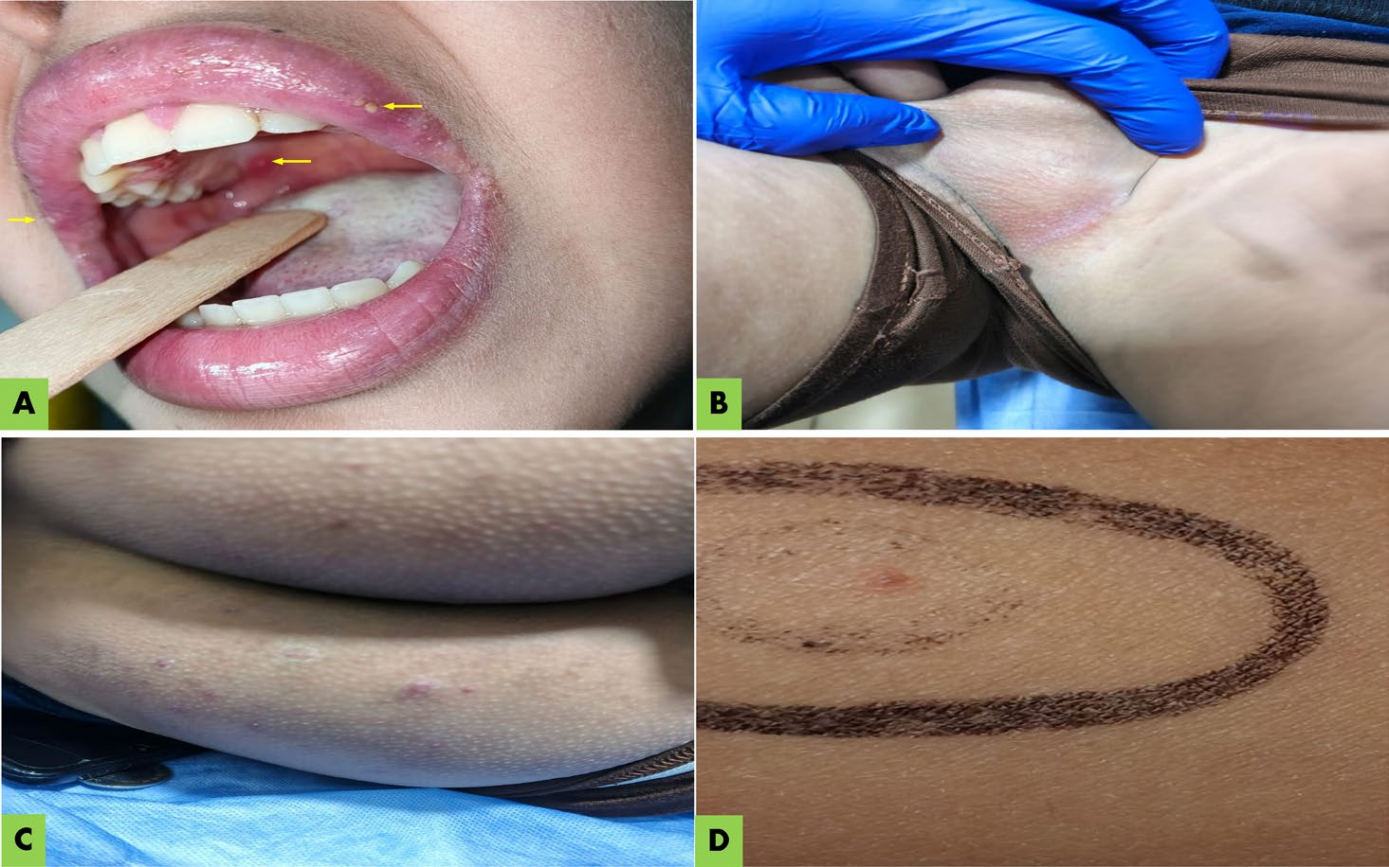
Composite clinical features of mucocutaneous‐predominant pediatric Behçet's disease (A) Intraoral photograph showing two aphthous ulcers, including a larger lesion on the posterior buccal mucosa/upper gingivobuccal sulcus adjacent to the maxillary molars and a smaller lesion on the inner upper lip. Yellow arrows indicate the aphthous ulcers. (B) Inguinal photograph showing clustered papulopustular lesions on a background of post‐inflammatory hyperpigmentation. (C) Photograph showing scattered erythematous papules over the buttock region. (D) Positive pathergy test on the volar forearm, showing a localized erythematous papule with a central punctate lesion at 24–48 h.

Examination showed two aphthous ulcers, one on the inner upper lip and another on the posterior buccal mucosa/upper gingivobuccal sulcus adjacent to the maxillary molars (Figure 1A). The inguinal region showed grouped papulopustular lesions on a background of hyperpigmentation (Figure 1B), and scattered erythematous papules were present over the buttocks (Figure 1C). Musculoskeletal examination showed tenderness of both knees without effusion, deformity, or restriction of movement. Mild right upper quadrant tenderness and bilateral tender cervical lymphadenopathy were also noted. No other abnormal findings were identified on systemic examination.

## 3 Differential Diagnosis, Investigations, and Treatment

The differential diagnosis in a child with recurrent oral and genital ulceration includes infections, inflammatory bowel disease, celiac disease, nutritional deficiencies, cyclic neutropenia, erythema multiforme, connective tissue disease, reactive arthritis, and autoinflammatory syndromes [2, 5]. In adolescents, genital ulceration also requires consideration of venereal infection when clinically relevant [4]. In this patient, the combined mucocutaneous pattern, systemic inflammatory markers, and positive pathergy reaction made isolated recurrent aphthous stomatitis less likely, while the negative autoimmune serology and non‐supportive celiac screening reduced support for some alternative explanations. In addition, infection‐related and safeguarding concerns were clinically reviewed during the case assessment, and the overall pattern favored a non‐infectious inflammatory disorder.

In view of the recurrent oral and genital ulceration, papulopustular skin lesions, and arthralgia, Behçet's disease was suspected. A forearm pathergy test was positive, with a well‐demarcated erythematous papule and a central punctate lesion developing at the needle‐prick site after 24–48 h (Figure 1D). Relevant investigations are summarized in Table 1, and the clinical course is outlined in Table 2. Laboratory evaluation showed an elevated erythrocyte sedimentation rate, mild leukocytosis, and thrombocytosis. Renal and liver screening tests, including bilirubin and routine electrolytes, were within normal limits on the available panel. Hemoglobin was normal at 13.7 g/dL, while MCV was 74.4 fL, consistent with isolated mild/borderline microcytosis without anemia. Ferritin, iron studies, and hemoglobinopathy screening results were not available in the records reviewed for this report. Therefore, a possible iron deficiency or thalassemia trait could not be excluded. However, this hematologic finding was considered concurrent. It did not account for the overall Behçet‐compatible phenotype, including recurrent oral and genital ulceration, papulopustular lesions, arthralgia, and a positive pathergy response. HLA‐B*51 was negative, and antinuclear antibody and extractable nuclear antigen testing were negative.

**TABLE 1:** Key diagnostic assessment and selected investigations.

Assessment	Result	Interpretation
Core clinical features	Recurrent oral aphthous ulcers, episodic painful scrotal ulcers, papulopustular skin lesions, and arthralgia	Mucocutaneous‐predominant presentation compatible with pediatric Behçet's disease
Family history	No known family history of Behçet's disease, recurrent oral/genital ulceration, or inflammatory bowel disease	No supportive family history identified
Infection/safeguarding review	No history suggestive of sexual exposure or abuse; overall pattern favored a non‐infectious inflammatory disorder	Reduced suspicion for STI‐related genital ulceration
Pathergy test	Positive at 24–48 h	Supportive bedside finding
CBC/inflammatory markers	Hemoglobin 13.7 g/dL; MCV 74.4 fL; WBC 13.87 × 10^3^/μL; platelets 415 × 10^3^/μL; ESR 43 mm/h	Isolated mild/borderline microcytosis without anemia; inflammatory activity supported by leukocytosis, thrombocytosis, and elevated ESR
Microcytosis evaluation	Ferritin, iron studies, and hemoglobinopathy screening results were not available in the records reviewed for this report	Isolated mild/borderline microcytosis without anemia; iron deficiency or thalassemia trait could not be excluded because ferritin, iron studies, and hemoglobinopathy screening results were not available in the records reviewed
Renal/liver profile	Creatinine 0.49 mg/dL; BUN 11.3 mg/dL; ALT 16.5 U/L; AST 20.2 U/L; ALP 156 U/L	No major renal or hepatic abnormality on the available panel
Autoimmune/supportive tests	HLA‐B*51 negative; ANA negative; ENA panel negative	Negative findings did not exclude Behçet's disease, and reduced support for some mimics
Celiac screening	tTG‐IgA 1.6 U/mL	Not supportive of celiac disease
Ophthalmology	No uveitis on slit‐lamp and dilated fundus examination	No ocular involvement identified on available ophthalmologic assessment
Gastroenterology evaluation	Gastroenterology review was unremarkable; no significant abnormality was identified on the available assessment	No significant gastrointestinal involvement was identified on the available evaluation
Follow‐up status	Sustained clinical improvement on colchicine, with no recurrence of genital ulceration reported, no major oral ulcer flare, persistent improvement in papulopustular lesions, and marked improvement in arthralgia	Supports stable early disease control during current follow‐up

*Note:* Routine electrolytes and bilirubin were within normal limits on the available panel.

**TABLE 2:** Relative timeline of presentation, assessment, treatment, and follow‐up.

Time point	Clinical course
2 years before the current assessment	Pulmonary hydatid cyst treated successfully; this prior condition was considered clinically incidental to the later Behçet‐compatible presentation
> 12 months before presentation	Recurrent painful oral ulcers began approximately monthly
Same period	Episodic scrotal ulceration, papulopustular skin lesions, intermittent arthralgia, fatigue, tender cervical lymphadenopathy, and intermittent abdominal discomfort with nausea and occasional diarrhea
Initial assessment	Oral aphthae, inguinal papulopustular lesions, buttock papules, knee tenderness, cervical lymphadenopathy; Behçet's disease was suspected clinically
24–48 h after pathergy testing	Positive pathergy response documented
Initial diagnostic work‐up	CBC, ESR, renal/liver profile, HLA‐B*51, ANA/ENA, and celiac screening were reviewed; ferritin, iron studies, and hemoglobinopathy screening results were not available in the records reviewed for this report
Start of treatment	Topical corticosteroids for oral/genital ulcers, supportive care, and colchicine 0.5 mg twice daily initiated
~3 weeks	No recurrence of genital ulceration, no full oral flare, clear skin improvement, improved arthralgia, and gastrointestinal symptoms partially improved without alarm features
~3–4 weeks	Ophthalmology review showed no uveitis; gastroenterology review arranged
~4 weeks	Repeat complete blood count, renal function tests, hepatic parameters, bilirubin, and routine electrolytes remained within normal limits; continued clinical improvement without new mucocutaneous flare
Subsequent follow‐up visits	Continued clinical improvement on colchicine, with no recurrence of genital ulceration reported, no major oral ulcer flare, sustained skin improvement, and marked improvement in arthralgia. Subsequent available safety monitoring, including complete blood count, renal function, and hepatic parameters, remained within normal limits
Latest follow‐up (April 2026)	The patient remained clinically well, with sustained mucocutaneous improvement, normal complete blood count, renal function, and hepatic parameters on available safety monitoring, reassuring ophthalmologic follow‐up, and unremarkable gastroenterology evaluation

Topical treatment was initiated for the oral and genital ulcers, consisting of triamcinolone acetonide 0.1% oral paste and clobetasol propionate 0.05% ointment, along with supportive measures, including a chlorhexidine mouth rinse and nonsteroidal anti‐inflammatory drugs as needed. Colchicine 0.5 mg twice daily was then initiated and was well tolerated.

## 4 Conclusion and Results (Outcome and Follow‐Up)

At approximately 3 weeks, there had been no recurrence of genital ulceration, no full oral ulcer flare, clear improvement in the papulopustular lesions, and improvement in arthralgia with minimal analgesic use. Gastrointestinal symptoms had partially improved, and no alarm features were reported. Ophthalmologic evaluation, including slit‐lamp and dilated fundus examination, showed no evidence of uveitis. A gastroenterology review was arranged because of the intermittent abdominal pain and diarrhea.

At approximately 4 weeks, repeat safety monitoring included complete blood count, renal function tests (creatinine and blood urea nitrogen), hepatic parameters (ALT, AST, alkaline phosphatase, and bilirubin), and routine electrolytes; all remained within normal limits on the available follow‐up assessment. The patient remained clinically improved, with no new mucocutaneous flare, and continued surveillance was planned for possible ocular, gastrointestinal, neurologic, or vascular involvement.

At subsequent follow‐up visits, the patient continued colchicine with sustained clinical improvement. No recurrence of genital ulceration was reported during follow‐up. There was no major oral ulcer flare; the papulopustular lesions remained clearly improved, and arthralgia was markedly reduced. Subsequent available safety monitoring, including complete blood count, renal function, and hepatic parameters, remained within normal limits.

At the most recent follow‐up in April 2026, the patient remained clinically well, with sustained mucocutaneous improvement and no evidence of ocular involvement on available follow‐up assessment. Gastroenterology evaluation was unremarkable, and no significant gastrointestinal involvement was identified on the available evaluation. Continued follow‐up was advised because pediatric Behçet's disease may evolve over time.

This case highlights a mucocutaneous‐predominant presentation of pediatric Behçet's disease in a child with recurrent oral and genital ulceration, papulopustular lesions, arthralgia, and a supportive positive pathergy test. Sustained clinical improvement during follow‐up supports the practical value of early recognition and first‐line treatment for mild pediatric disease. Still, continued surveillance remains essential, as ocular, gastrointestinal, neurologic, or vascular manifestations may emerge over time.

## 5 Discussion

This case describes pediatric Behçet's disease presenting with recurrent oral aphthae, episodic genital ulceration, papulopustular skin lesions, arthralgia, and a positive pathergy test. None of these individual findings is unusual in Behçet's disease; the clinical value of the case lies instead in their coherent combination in a child, because pediatric disease often presents stepwise and incompletely, which can delay recognition [4, 6].

Behçet's disease remains a clinical diagnosis because no single laboratory or histopathologic test is diagnostic on its own [7]. In this patient, the diagnostic argument is strengthened by mapping the phenotype to established classification frameworks. Using the International Criteria for Behçet's Disease, recurrent oral aphthosis, genital aphthosis, and skin lesions, together with a positive pathergy test, meet the classification threshold [8]. The pediatric PEDBD criteria are also relevant because they were developed for Behçet's disease in childhood, where manifestations may be incomplete early in the course [6]. In this case, the oral, genital, and skin domains provide the minimum number of distinct categories required by the pediatric framework.

The pathergy test was a particularly useful supportive finding. It reflects an exaggerated inflammatory response to minor trauma and remains clinically informative when interpreted in the setting of a compatible phenotype, even though reported positivity rates vary across populations [4, 9]. The positive pathergy reaction in this patient, therefore, added bedside support to a diagnosis already suggested by the overall mucocutaneous pattern.

Published pediatric series consistently show that oral ulceration is often the earliest manifestation and that mucocutaneous involvement is commonly dominant at presentation, whereas ocular, gastrointestinal, neurologic, and vascular manifestations may emerge later [6, 10, 11]. This pattern is consistent with the present case and supports its interpretation as an early mucocutaneous‐predominant form of pediatric Behçet's disease rather than an isolated recurrent ulcerative disorder.

Although HLA‐B51 is a recognized susceptibility marker, it is neither necessary nor sufficient for diagnosis, and a negative result does not exclude the disease [16]. In daily practice, the clinical phenotype and supportive bedside findings are often more informative than genetic testing in individual pediatric cases. The positive pathergy test was therefore more diagnostically useful in this patient than the negative HLA‐B51 result.

Although this presentation was mucocutaneous‐predominant, structured surveillance remains essential because major‐organ involvement may emerge later and is responsible for much of the long‐term morbidity. Ocular disease is especially important because it may threaten vision over time, particularly in male patients [12]. Gastrointestinal involvement can present with abdominal pain and diarrhea, and may progress to more serious ulcerative disease in some patients [13]. Neurologic and vascular disease also require attention because severe outcomes in Behçet's disease are largely linked to central nervous system and major‐vessel involvement [14, 17]. In the present case, the absence of uveitis on slit‐lamp and dilated fundus examination was reassuring, but it does not remove the need for continued ophthalmic follow‐up. Similarly, the initial gastrointestinal symptoms justified further evaluation, but the subsequent gastroenterology assessment was unremarkable on the available follow‐up.

Structured surveillance remains important because ocular, gastrointestinal, neurologic, and vascular involvement may emerge during the disease course and account for clinically important morbidity in Behçet's disease [12, 13, 15, 17]. In under‐resourced or community settings, however, multispecialty follow‐up may be limited by restricted access to subspecialists and risk of loss to follow‐up. A pragmatic approach includes baseline ophthalmologic assessment when feasible, symptom‐based review at primary‐care visits, family counseling on red‐flag symptoms, and targeted referral based on evolving clinical features.

The early response to treatment also supports a phenotype‐based management approach. Current recommendations support topical corticosteroids for active oral and genital ulcers and colchicine as a first‐line systemic option for recurrent mucocutaneous disease [15]. After treatment was started, this patient showed no recurrence of genital ulceration, no full oral ulcer flare, clear improvement in papulopustular lesions, and improvement in arthralgia within a few weeks. That response is biologically plausible in a disorder in which neutrophil‐driven inflammation contributes to mucocutaneous activity [18]. More intensive treatment would generally be reserved for refractory mucocutaneous disease or organ‐threatening involvement [15].

The differential diagnosis in a child with recurrent oral and genital ulceration includes infections, inflammatory bowel disease, celiac disease, nutritional deficiencies, cyclic neutropenia, erythema multiforme, connective tissue disease, reactive arthritis, and autoinflammatory syndromes [2, 5]. In adolescents, genital ulceration also requires consideration of venereal infection when clinically relevant [4]. In this patient, the combined mucocutaneous pattern, systemic inflammatory markers, and positive pathergy reaction made isolated recurrent aphthous stomatitis less likely, while the negative autoimmune serology and non‐supportive celiac screening reduced support for some alternative explanations. In addition, infection‐related and safeguarding concerns were clinically reviewed during the case assessment, and the overall pattern favored a non‐infectious inflammatory disorder.

This report has limitations. Despite sustained clinical improvement during follow‐up, pediatric Behçet's disease may evolve over time, and continued observation is needed to assess relapse pattern and later organ involvement. The gastroenterology evaluation was reassuring based on the available assessment, but longitudinal surveillance remains appropriate if new symptoms emerge. Another limitation is that the isolated mild/borderline microcytosis was not fully characterized by ferritin, iron studies, or hemoglobinopathy screening in the records reviewed for this report; therefore, concurrent iron deficiency or thalassemia trait could not be excluded.

## Author Contributions

**Fares Basel Abu Taha:** conceptualization, methodology, investigation, writing – original draft, writing – review and editing. **Alaweya A. Ofash:** conceptualization, methodology, investigation, writing – original draft, writing – review and editing. **Osama S. Abbadi:** conceptualization, methodology, investigation, writing – original draft, writing – review and editing. **Abdelrahman M. A. Abukanna:** supervision, visualization, resources, writing – original draft, writing – review and editing. **Mohammed Moizulddin Khan:** validation, visualization, resources, writing – review and editing, data curation. **Mahir Mohammed:** investigation, supervision, resources, writing – original draft, writing – review and editing. **Faris Abdon:** conceptualization, methodology, validation, visualization, writing – original draft, writing – review and editing. **Amani M. Nabri:** supervision, resources, validation, writing – original draft, writing – review and editing. **Adil Khalil Hussien Khalil:** validation, visualization, resources, writing – original draft, writing – review and editing.

## Funding

The authors have nothing to report.

## Ethics Statement

The authors have nothing to report.

## Consent

Written informed consent was obtained from the patient's legal guardian for publication of this case report and any accompanying images. Given the patient's age, the consent process was reviewed for pediatric assent documentation; a separate written assent document was not available in the records reviewed. A copy of the written guardian consent is available for review by the Editor‐in‐Chief of this journal.

## Conflicts of Interest

The authors declare no conflicts of interest.

## Data Availability

All data supporting the findings of this case report are included in this published article and its tables and figure. Additional de‐identified information can be made available by the corresponding author upon reasonable request, where permitted.
